# Combination of a MIP3α-antigen fusion therapeutic DNA vaccine with treatments of IFNα and 5-Aza-2’Deoxycytidine enhances activated effector CD8+ T cells expressing CD11c in the B16F10 melanoma model

**DOI:** 10.21203/rs.3.rs-3243336/v2

**Published:** 2024-08-20

**Authors:** Kaitlyn Fessler, Jiaqi Zhang, Avinaash K. Sandhu, Yinan Hui, Aakanksha R. Kapoor, Samuel K. Ayeh, Styliani Karanika, Petros C. Karakousis, Richard B. Markham, James T. Gordy

**Affiliations:** Johns Hopkins Bloomberg School of Public Health; Johns Hopkins Bloomberg School of Public Health; Johns Hopkins Bloomberg School of Public Health; Johns Hopkins Bloomberg School of Public Health; The Johns Hopkins Hospital; The Johns Hopkins Hospital; The Johns Hopkins Hospital; The Johns Hopkins Hospital; Johns Hopkins Bloomberg School of Public Health; Johns Hopkins Bloomberg School of Public Health

**Keywords:** MIP3α/CCL20, IFNα/Interferon alpha/Type I interferon, 5-Aza-2’-Deoxycytidine/Decitabine/Dacogen, CD11c, Tumor microenvironment, CD11c+ CD8+ T cell, DNA Therapeutic Cancer Vaccine

## Abstract

Previous studies in the B16F10 mouse melanoma model have demonstrated that combining a DNA vaccine comprised of regions of gp100 and tyrosinase-related protein 2 fused to Macrophage-inflammatory protein 3-alpha (MIP3α) with recombinant Interferon alpha (IFN) and 5-Aza-2’-Deoxycytidine (5Aza) treatments resulted in significantly greater anti-tumor activity and immunogenicity in the tumor microenvironment (TME). This brief report details that the combination of vaccine with treatments IFN and 5Aza results in both the upregulation of genes expressing CD11c-interacting proteins and an increase in the TME of a distinct CD11c+ CD8+ T cell population. This cell population correlates with tumor size, is primarily comprised of effector or effector memory T cells, and has a more robust response to ex vivo stimulation as compared to CD11c− CD8+ T cells as measured by surface activation markers 4-1BB (CD137) and KLRG1 (Killer cell lectin-like receptor G1) and intracellular IFNγ production. In conclusion, this combination therapy results in greater presence of highly active effector CD8+ T-cells expressing CD11c in the TME that correlate with and are likely primary contributors to treatment efficacy.

## Introduction

**Figure 1:**
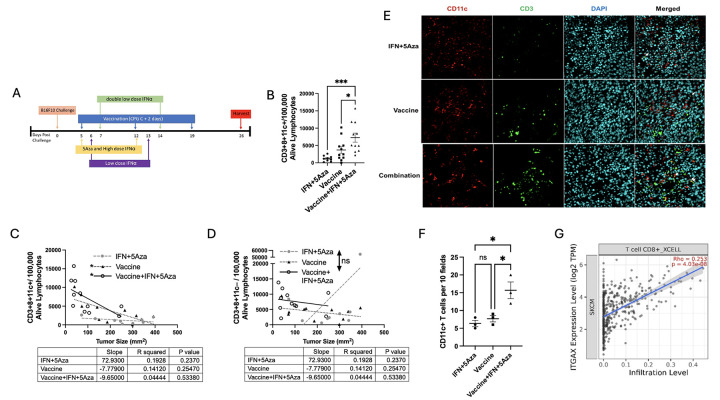
CD3+CD8+CD11c+ cells in the tumor microenvironment (TME). A) Timeline for treatments for all experiments in this study. B) The number of CD3+CD8+CD11c+ cells per 100,000 alive lymphocytes between treatment groups. C) The correlation of CD3+CD8+CD11c+ cells and D) CD3+CD8+CD11c− cells to tumor size stratified by group. E) Representative immunofluorescence staining images of CD11c+ cells and T cells. Cells were stained by antibodies to CD3 (green), CD11c (red), and DAPI (cyan). Yellow: colocalization of CD3 and CD11c labeled by both antibodies, shown by white arrows. x400 total magnification. F) Counts of CD11c+ T cells per 10 fields across the analyzed microscopy samples. G) Correlation analysis of publicly available datasets comparing CD11c (ITGAX) expression level and CD8+ T cell infiltration in SKCM. (n= 471). Data for panels B-D were combined from three independent experiments with sample size ranging from 9–11 total animals per group. Microscopy analyzed three randomized animals per group and representative images are shown. *p<0.05, **p<0.01, ***p<0.001.

Cancer therapeutic vaccines combined with immunotherapies and chemotherapies have garnered interest due to their potential synergistic effects[1]. Previous preclinical studies have demonstrated that DNA vaccines comprising melanoma antigen(s) fused to MIP3α delayed tumor growth, prolonged survival, and enhanced T-cell immunity in the B16F10 melanoma model[2–4]. MIP3α vaccine fusions have been shown to target antigen(s) to immature dendritic cells (iDC), with presentation through MHC class I and II[5, 6]. Here, clinically-relevant antigens gp100_25–235_ and tyrosinase-related protein 2 (Trp2_170–269_)[7] are fused to MIP3α. When MIP3α-Gp100-Trp2 vaccine adjuvanted with CpG type C (henceforth referred to as “vaccine”) was combined with the drug treatments low-dose 5Aza-2’Deoxycytidine (5Aza) and a series of high and low doses of recombinant IFNα (IFN) (Fig. 1A), significantly greater anti-tumor activity was achieved, including increased dendritic cell (DC) and CD8+ T-cell infiltration into the tumor microenvironment (TME)[3, 8].

CD11c is a type I transmembrane protein forming part of the complement receptor 4 and has been shown to play a role in phagocytosis, cell migration, cytokine production, and T-cell proliferation. A subset of CD8+ T cells expressing CD11c+ have been described in various tissues, infections, and cancers[9–11] with the ability to become suppressor or effector cells[10, 12, 13]. The current study shows that levels of CD8+ T cells expressing CD11c are increased in the TME with combination treatment and are correlated with tumor size in vaccinated groups. To our knowledge, this study is the first to show the presence of CD8+CD11c+ T cells in a tumor-associated-antigen based therapeutic cancer vaccine system, and the data suggest that these cells are in higher numbers, are more robust, and are more potent with the combination treatments compared to vaccine alone or treatments alone. These cells are therefore likely to be critical components in the tumor suppression observed with the therapeutic regimen we have employed against the B16F10 melanoma.

## Materials and Methods

### Animals

6–12-week-old female C57BL/6 (Charles River, Wilmington, MA) mice were challenged with a lethal dose of B16F10 melanoma (5×10^4^ cells, >95% viability) administered intradermally on the mouse flank on day 0 of therapy[2, 3, 8]. Tumor size was recorded by calipers every 1–3 days as square mm (L × W). Mice were monitored in accordance with IACUC protocols and were removed from the study once tumo diameterr>1cm or if mice showed signs of distress.

### Vaccines and Therapies

Vaccine antigen is the MIP-3α-Gp100-Trp2 (tyrosinase-related protein 2) DNA construct in the pCMVe mammalian expression plasmid[8]. Therapy including vaccine, recombinant mouse interferon alpha-A (IFNα, R&D Systems, Inc. Minneapolis, MN), and InSolution^™^ 5 Aza 2′-deoxycytidine (5Aza, CalBiochem^®^, MilliporeSigma, Burlington, MA) has been detailed in prior publications[8] and is outlined in Figure 1A.

### Flow Cytometry

Tumor cell and splenic suspensions were prepared as previously described [2, 3]. The Attune^™^ NxT (Thermo Fisher Scientific, Waltham, MA) flow cytometer was utilized. Data were analyzed by FlowJo Software (FlowJo, LLC Ashland, OR) or Attune NxT Software v3.2.1. Tumors smaller than 25mm^2^ were not analyzed. Supplemental Figure 1 outlines gating structure and stimulation procedures[3]. Gates were formulated using full-minus-one (FMO) staining controls.

### Immunofluorescence Staining

Tumor tissues were collected at the time of harvest and were immediately fixed in 10% neutral-buffered formalin for 48 hours and were then processed, embedded into paraffin (FFPE), and cut onto slides. The slides were blocked, stained with αCD3 antibody, washed, stained with AF488 secondary antibody, washed, and repeated for αCD11c antibody and AF647 secondary. The nuclei were counterstained with DAPI and mounted. Three slides were blindly selected from each group for analysis. Images were acquired across ten random fields per slide under the 40x objective using a Leica THUNDER computational clearing widefield microscope through LAS X software (Leica Microsystems, Wetzlar, Germany).

### Database Collection and Statistics

The correlations between CD11c (ITGAX) expression and infiltrating immune cell subsets in the tumor microenvironment were assessed through TIMER 2.0 web server (http://timer.cistrome.org/) and the xCell algorithm. Purity-adjusted Spearman’s rho and statistical significance were used to evaluate correlations. Datasets were tested for normality by D’Agostino & Pearson test and followed by by one-way ANOVA with Tukey’s or Dunn’s multiple comparison test for multiple groups, or by unpaired Student’s T test or Mann-Whitney tests for two groups, Scatter plots were analyzed by linear regression with Pearson correlation coefficient test. Grouped experiments were analyzed by 2-way ANOVA with Sidak’s multiple comparison test. Sample sizes are defined in the figure legends. GraphPad Prism^™^ 10 (GraphPad Software, Inc. San Diego, CA) was utilized for statistical analyses and figure creation. Error bars represent the estimation of the standard error of the mean and midlines the group mean. α≤0.05

## Results

### CD8+11c+ T cells in the TME

In our model combining vaccine with IFN and 5Aza (Fig. 1A), the CD8+CD11c+ T-cell population was significantly higher following combination therapy vs. vaccine alone (p=0.0271) or IFN+5Aza (p=0.0007) (Fig. 1B). Of note, when stratified across groups, the presence of the CD11c+ CD8+ T-cell subpopulation was also correlated with tumor size in vaccinated groups, whereas CD11c− CD8+ T cell numbers were not correlated (Fig. 1C–D). Complementing the flow cytometry data, the presence and distribution of T cells in the intratumoral areas were determined by immunofluorescence microscopy (Fig. 1E). A significantly higher number of CD11c+ T cells were observed in the group that received combination therapy as compared with IFN+5Aza (P=0.0118) or vaccine alone (P=0.0234) (Fig. 1F). The relationship between CD11c (ITGAX) expression and CD8+ T cell infiltration was further explored by analyzing publicly available datasets of melanoma patients (Fig. 1G). The results suggest a significant positive correlation between CD11c expression and the overall infiltration level of CD8+ T cells (rho= 0.23, P= 4.03e-08).

### Memory subtyping of CD8+11c+ T cells in the TME

**Figure 2:**
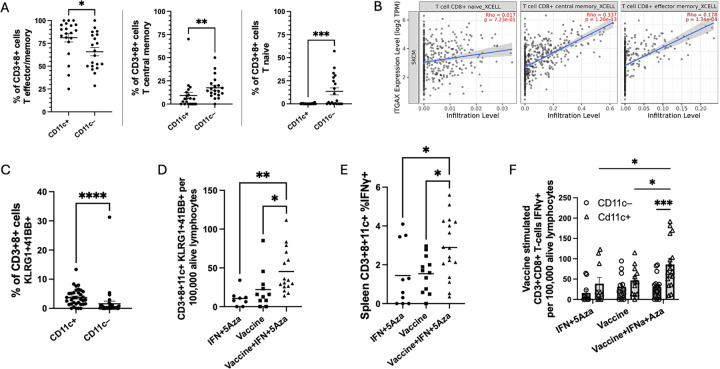
Analysis of CD3+CD8+CD11c+ cell memory and activation markers: A) Effector/memory status of CD8+ T cells in the TME stratified by CD11c presence at day 26 of our mouse model. B) Correlation analysis of publicly available datasets comparing CD11c (ITGAX) expression level and CD8+ T cell memory subsets’ infiltration in SKCM. (n= 471). C-F utilize ex-vivo stimulated splenocytes from day 26 of the mouse model. C) Percentage of CD11c+ or CD11c− CD8 T-cells expressing KLRG1 and 41BB. D) The number of KLRG1+41BB+ CD11c+ CD8 T cells post-stimulation across the groups. E) Percentage across groups of CD11c+ CD8+ T cells expressing IFNy post-stimulation. F) Number of CD11c+ and CD11c− CD8 T cells expressing IFNy post-stimulation across the groups and stratified by CD11c positivity. Samples are derived from three independent experiments with sample size ranging from 8–17 total animals per group. *p<0.05, **p<0.01, ****p<0.0001.

The overall phenotype of CD3+CD8+CD11c+ cells infiltrating into the TME was explored, independent of treatment group. CD3+CD8+CD11c+ cells were categorized into three groups based on CD44 and CD62L expression: CD62L+CD44+ central memory (Tcm); CD62L−CD44+ effector and effector memory (Teff/em); and CD62L+CD44− naive (Tnaive). CD8+CD11c+ cells had a significantly greater proportion of Teff/em cells compared to their CD11c− counterparts (p=0.01) and significantly smaller proportions of Tnaive (p<0.001) and Tcm (p=0.001) cells (Fig. 2A). Infiltration scores from publicly available human datasets also show that CD11c is positively correlated with the infiltration levels of CD8+ T effector memory cell subtypes (rho= 0.178, P= 1.34e-04), but not CD8+ naïve T cells (rho= 0.017, P= 0.723) (Fig. 2B). Surprisingly, a positive correlation is also observed between CD11c and infiltrating CD8+ central memory T cells (rho= 0.337, P= 1.26e-13) (Fig. 2B), which differs from our mouse model data (Fig. 2A).

### Activation Markers of CD8+11c+ T cells in the Spleen

Cryopreserved splenocytes were analyzed to further assess the subset of CD11c+ CD8+ T cells for markers of effector function and activation: 4-1BB, Killer cell lectin-like receptor G1 (KLRG1), and IFNγ. Following ex-vivo stimulation with vaccine peptides, we show that co-expression of KLRG1 and 4-1BB was significantly higher in CD8+CD11c+ T cells (p<0.001) compared to CD11c− cells (Fig.2C). Further, when stratified by treatment group, expression of 41BB and KLRG1 on the surface of CD8+CD11c+ T cells was increased significantly following combination therapy relative to treatment with IFN+5Aza (p=0.0020) or vaccine alone (p=0.0250) (Fig. 2D). Using intracellular staining methods, IFNγ expression was greater in the combination group compared to the IFN+5Aza group (p= 0.0393) and the vaccine group (p= 0.0426) (Fig. 2E). Additionally, after stratifying the CD8+ T cells by CD11c expression, the CD11c+ population carries the phenotype of higher cytokine production in the combination group compared to IFNα+5Aza (p=0.0110) and vaccine (p= 0.0319) (Fig. 2F). Moreover, the CD11c+ cells had a significantly greater proportion of cells expressing IFNγ in the combination group compared to their CD11c− counterparts (p=0.0019) (Fig. 2F).

## Discussion

The current study provides evidence that CD8+CD11c+ T cells are increased with combination therapy, are correlated with tumor size (Fig. 1), and express more markers associated with activation and effector function (Fig. 2). 4-1BB is hypothesized to be a necessary costimulatory marker for CD8+CD11c+ T cells and is known to promote T-cell survival and enhance expression of cytokines such as IFNγ[14]. KLRG1 is induced on highly cytolytic, short-lived effector CD8+ T cells and is hypothesized to play a role in CD8+CD11c+ T-cell activity [15]. Interestingly, the majority of the IFNγ production induced by our vaccination protocol is attributable to the CD8+CD11c+ and not the CD8+CD11c− T-cell population (Fig. 2F). Some studies have pointed to stronger regulatory activity of this cell poopulation [10], but others have shown, similar to results here, that CD8+CD11c+ T cells aid in successful antitumoral responses via enhanced effector functions [9], including direct anti-tumor cytotoxicity[13]. Our studies have identified an immunization protocol that is particularly effective in activating this T cell population.

Analysis of human melanoma trials from publicly available databases show that intratumoral CD11c expression correlates with increased CD8+ T-cell infiltration (Fig. 1F), especially effector/memory subtypes (Fig. 2B), and that patients with more expression have improved survival outcomes (Supp Fig. 3) and better responses to immune checkpoint inhibitor therapy (Supp Fig. 4). We hypothesize that the stronger Tcm correlation in patients compared to our results in the mouse model may be attributable to the more prolonged time frame of melanoma persistence in the clinical setting. Importantly, clinical data supports the interaction of CD11c with CD8+ T-cells and with improved outcomes in melanoma.

Our results show that vaccine-induced, activated, IFNγ-secreting, effector/memory CD8+CD11c+ T cells significantly correlate to the antitumoral effect elicited by our combination therapy and are dependent on the combination of vaccine and drug treatments, as removing either arm limits the induction of these cells. These results combined with the literature [9, 13–15] suggest that the vaccine-induced CD8+CD11c+ T cells are primary effectors of the anti-tumor immune response. Future studies will further delineate whether these T-cells 1) are relevant to other tumor models; 2) traffic into the tumor or develop at the site 3) vary in number and potency over time; 4) interact with other cells in the tumor; and 5) have direct anti-tumor cytotoxicity.

## Data Availability

Datasets utilized in the study are supplied in the Supplemental Data File. Further information is available by the corresponding authors upon reasonable request.

## Abbreviations

MIP3α: Macrophage inflammatory protein 3-alpha (aka CCL20)
IFN: Interferon Alpha
5Aza: 5’-Aza-2’-deoxycytidine
TME: Tumor Microenvironment
iDC: immature dendritic cell
Trp2: Tyrosinase-related protein 2
Tcm and Tem: T cell central and effector memory, respectively
KLRG1: Killer cell lectin-like receptor G1
